# Retraction: Indirect Recruitment of a CD40 Signaling Pathway in Dendritic Cells by B7-DC Cross-Linking Antibody Modulates T Cell Functions

**DOI:** 10.1371/annotation/36ac4b2c-cf27-41d9-90e2-e5d58d307896

**Published:** 2010-03-03

**Authors:** Suresh Radhakrishnan, Rosalyn Cabrera, Kristina M. Bruns, Virginia P. Van Keulen, Michael J. Hansen, Sara J. Felts, Larry R. Pease

An investigation by the Mayo Clinic has determined that one of the researchers in Professor Pease's laboratory at the Mayo Clinic, Dr. Suresh Radhakrishnan, tampered with another investigator's experiment with the intent to mislead toward the conclusion that the B7-DCXAb reagent has cell activating properties. Using blinded protocols, experiments were done to see if the results based on this reagent could be replicated. Specifically, the repeat experiments examined the activation of dendritic cells, activation of cytotoxic T cells, induction of tumor immunity, modulation of allergic responses, breaking tolerance in the RIP-OVA diabetes model, and the reprogramming of Th2 and T regulatory cells. In no case did these repeat studies reveal any evidence that the B7-DCXAb reagent had the previously reported activity. The authors of this paper therefore wish to retract this paper because of the inability to reproduce key aspects of the studies and hence the results in them cannot be considered reliable.

